# Alterations in the Temporomandibular Joint Space Following Orthognathic Surgery Based on Cone Beam Computed Tomography: A Systematic Review

**DOI:** 10.3390/jcm14207239

**Published:** 2025-10-14

**Authors:** Marta Szcześniak, Julien Issa, Aleksandra Ciszewska, Maciej Okła, Małgorzata Gałczyńska-Rusin, Marta Dyszkiewicz-Konwińska

**Affiliations:** 1 Department of Diagnostics, Poznan University of Medical Sciences, 60-812 Poznan, Poland; 2 Department of Maxillofacial Surgery, Poznan University of Medical Sciences, 60-812 Poznan, Poland; 3 Doctoral School, Poznan University of Medical Sciences, 60-812 Poznan, Poland; 4 Department of Oral Radiology & Digital Dentistry, Academic Centre for Dentistry Amsterdam (ACTA), University of Amsterdam and Vrije Universiteit Amsterdam, Gustav Mahlerlaan 3004, 1081 Amsterdam, The Netherlands; 5 Department of Orthodontics and Temporomandibular Disorders, Poznan University of Medical Sciences, 60-812 Poznan, Poland

**Keywords:** temporomandibular joint, orthognathic surgical procedures, joint space width, cone-beam computed tomography

## Abstract

**Background/Objectives:** Orthognathic surgery represents a surgical modality for the correction of craniofacial skeletal deformities. These procedures help achieve stable occlusion and improve facial symmetry, which in turn enhances functional outcomes and overall quality of life. However, to date, no consensus has been reached regarding whether orthognathic surgery also induces changes in the relationship of articular surfaces within the temporomandibular joints (TMJs). The primary objective of this study was to conduct a systematic review of research evaluating joint space dimensions based on CBCT imaging performed before and after orthognathic surgery. **Methods:** A comprehensive literature search was carried out across four electronic databases: PubMed, Web of Science, Cochrane Library, and Scopus. Two independent reviewers screened titles and abstracts according to predefined inclusion criteria. Eligible studies were subjected to critical appraisal, and relevant data were systematically extracted and summarized in tabular form. **Results:** Fourteen studies published between 2010 and 2024 met the inclusion criteria. In all studies, CBCT-based joint space measurements were conducted at least twice once preoperatively and once postoperatively, across a total of 527 patients included in the review. **Conclusions:** The synthesized evidence suggests that orthognathic surgery produces measurable modifications in the spatial relationship of TMJ articular surfaces. Nonetheless, the clinical relevance of these alterations appears to be modulated by several variables, including the surgical technique employed and the patient’s individual adaptive capacity.

## 1. Introduction

Skeletal craniofacial deformities constitute a category of malocclusions resulting from aberrant dimensions and spatial relationships of the maxilla and mandible. Their management requires an interdisciplinary approach, typically integrating both orthodontic and surgical interventions [1]. These deformities compromise not only the aesthetic profile of the face but also masticatory function and may contribute to the onset of temporomandibular disorders (TMDs) or obstructive sleep apnea (OSA) [1]. An increasing number of patients with such anomalies are opting against orthodontic camouflage strategies in favor of combined orthodontic–surgical management, which remains the only etiologically based therapeutic choice. Craniofacial skeletal deformities may present in the sagittal plane, manifesting as retrognathia, retrognathism, prognathia, or prognathism, or in the coronal plane, as seen in laterognathia [1].

Surgical correction of these skeletal deformities falls within the domain of orthognathic surgery, comprising a range of procedures designed to re-establish normal proportions of the maxillomandibular complex and achieve optimal maxillomandibular relationships [2]. This is most commonly achieved through osteotomies of the maxilla and/or mandible, which enable repositioning of skeletal segments to improve both occlusal function and facial aesthetics [2,3].

In many cases, bimaxillary surgery is required to achieve a symmetrical, proportionate facial structure and stable occlusion. Among these, the Le Fort I osteotomy remains the most frequently performed maxillary orthognathic procedure [2], while the bilateral sagittal split osteotomy (BSSO) is the predominant mandibular approach [3,4]. In addition, alternative mandibular osteotomy techniques have been described, including intraoral vertical ramus osteotomy (IVRO) [5] and high oblique sagittal split osteotomy (HSSO) [6], although these approaches are less commonly utilized compared to the standard BSSO. Regardless of the specific surgical protocol employed, such interventions culminate in the establishment of a new occlusal relationship, along with all consequences in the functional dynamics of the stomatognathic system.

The temporomandibular joint (TMJ) is a bilaterally symmetrical articulation that enables coordinated mandibular movement [7]. Anatomically, it comprises three primary components: the condylar head of the mandible, the articular disc, and the mandibular fossa of the temporal bone [7]. Structurally, the joint is organized into two functional compartments—the superior compartment, situated between the temporal bone and the articular disc, and the inferior compartment, located between the articular disc and the mandibular condyle [7]. This biplanar arrangement enables complex mandibular motions, including rotation and translation [7].

It has been shown that alterations in occlusion, depending on the presence of additional psychological or behavioral factors, may influence the structural and functional characteristics of the temporomandibular joint [8]. Within a multifactorial and biopsychosocial framework, TMD may develop secondarily in the presence of additional contributing factors, such as tooth loss, malocclusion, or transient occlusal disturbances following dental procedures [8,9]. Chronic alterations in occlusion may lead to adaptive or pathological remodeling within the TMJ, potentially resulting in impaired function [10,11]. The degree to which various contributing factors affect the temporomandibular joint is further modulated by individual adaptive capacity and inherent anatomical predispositions [12].

This consideration raises a question: Do orthognathic surgical procedures, intended to restore optimal occlusal relationships, induce changes in the relationship of the articular surfaces in the temporomandibular joint? The aim of this review is to systematically analyze current evidence and determine whether orthognathic surgery induces modifications in the spatial relationships of the articular surfaces within the TMJ and to define the nature of these alterations.

## 2. Materials and Methods

### 2.1. Protocol and Registration

This systematic review was conducted in accordance with the Preferred Reporting Items for Systematic Reviews and Meta-Analyses (PRISMA) 2020 guidelines [13]. Following the database search, the review protocol was developed and registered on 3 August 2025 (CRD420251114762).

### 2.2. Eligibility Criteria

The eligibility criteria for this systematic review were established using the PECO (Population, Exposure, Comparator, and Outcome of interest) mnemonic. In addition, the availability of full text was also considered.

PECO Framework:

- Population (P): Patients qualified for orthognathic surgery (with or without orthodontic preparation).
- Exposure (E): Orthognathic surgical procedures, including Le Fort I osteotomy, BSSO, intraoral vertical ramus osteotomy (IVRO), and high oblique sagittal split osteotomy (HSSO).
- Comparator (C): Preoperative TMJ space measurements.
- Outcome (O): Quantitative changes in TMJ space, measured in the sagittal and/or coronal planes using CBCT.

The inclusion and exclusion criteria used to guide study selection are summarized in Table 1.

### 2.3. Data Sources

The literature search was conducted on 9 April 2025, across four electronic databases: PubMed, Web of Science, Cochrane Library, and Scopus. To ensure the inclusion of all relevant studies, additional sources were identified through manual screening of reference lists from included articles.

### 2.4. Search Strategy

A comprehensive search strategy was developed and tailored to the specific syntax and search functionalities of each database. The strategy incorporated a combination of Medical Subject Headings (MeSH) and keywords, including temporomandibular joint, TMJ, space, orthognathic surgery, Le Fort I, bilateral sagittal split osteotomy, intraoral vertical ramus osteotomy, surgical orthodontic treatment, and change. Boolean operators “AND” and “OR” were used to optimize search sensitivity and ensure broad inclusion of relevant studies. The complete search strategies for each database are presented in Supplementary Table S1. No filters, publication date limits, or language restrictions were applied during the search.

### 2.5. Study Selection

All search results were imported into Rayyan, a web-based platform designed for systematic review management [14]. Duplicate records were automatically identified using the built-in deduplication function and subsequently verified manually by one reviewer to ensure accuracy.

Prior to the screening process, a calibration meeting was held between the two reviewers to standardize the application of inclusion and exclusion criteria. A pilot screening of a subset of studies was conducted to ensure consistency in interpretation and decision-making.

Following deduplication, the remaining unique records were independently screened by two reviewers (M.S. and J.I.) based on their titles and abstracts. Studies that clearly did not meet the predefined eligibility criteria were excluded and documented. Full-text articles were retrieved for studies that met the inclusion criteria or for which eligibility remained uncertain after abstract screening. These full texts were further assessed independently by the reviewers using the predefined criteria. Any disagreements during the screening process were resolved through discussion, and if consensus could not be reached, a third reviewer was consulted.

### 2.6. Data Extraction

A standardized data extraction form was developed and piloted on a random subset of the included studies to assess clarity, completeness, and reviewer agreement. Based on this pilot testing, minor adjustments were made to improve usability and standardization.

Data extraction was performed independently by the two reviewers (M.S. and J.I.) using the finalized form. Any discrepancies between reviewers were discussed and resolved through consensus; unresolved conflicts were adjudicated by a third reviewer. To ensure accuracy, all extracted data were cross-verified.

The following information was extracted from each study: bibliographic details (author(s), year of publication, and study location), sample size, skeletal class, surgical procedure, timing of CBCT acquisition (preoperative and postoperative), preoperative and postoperative measurements in the sagittal and coronal planes, and reported *p*-values for statistical comparisons of measurements.

### 2.7. Risk of Bias and Quality Assessment

Given the design of the included studies, where changes in TMJ space are assessed pre- and post-orthognathic surgery, the risk of bias was evaluated using the NIH Quality Assessment Tool for Before–After (Pre–Post) Studies With No Control Group [15]. This tool is specifically designed to assess the internal validity of studies that evaluate outcomes before and after an intervention within the same group of participants [15].

Each study was independently assessed by two reviewers using the tool’s standardized criteria, and discrepancies were resolved through discussion or consultation with a third reviewer when necessary. The assessment considered aspects such as participant selection, consistency of the intervention, outcome measurement reliability, statistical analysis, and handling of missing data. Ratings for each criterion were recorded as Yes, No, Cannot Determine, Not Reported, or Not Applicable. The overall methodological quality of each study was determined based on the collective evaluation of these domains.

## 3. Results

### 3.1. Search Results

**Figure 1:**
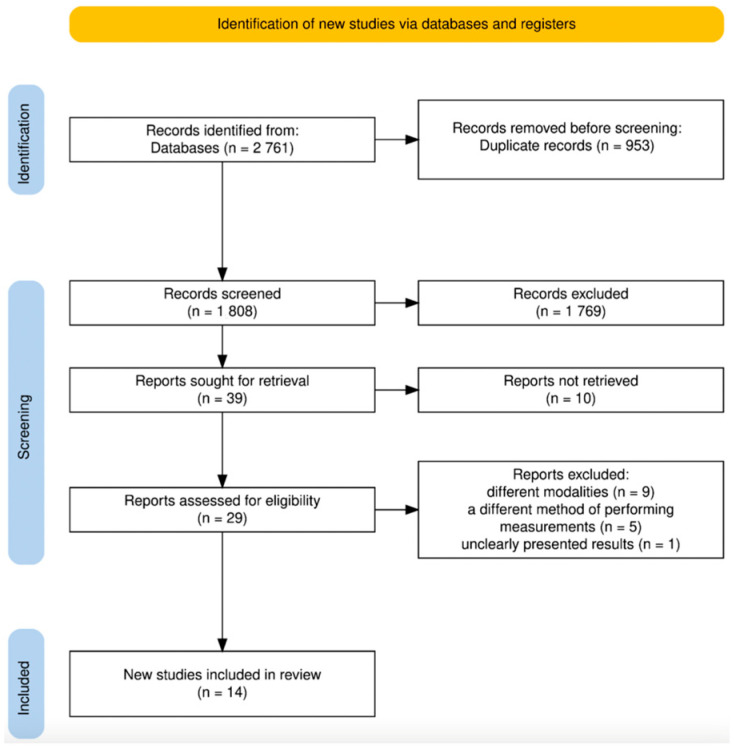
PRISMA flow diagram.

The outcomes of the systematic literature search are presented in the flow diagram (Figure 1) in accordance with the PRISMA guidelines [16]. A total of 2761 records were retrieved from the designated electronic databases. Following the removal of duplicate entries, 953 records were excluded. The remaining 1808 titles and abstracts were screened based on predefined inclusion and exclusion criteria, yielding 39 articles deemed eligible for full-text evaluation. After a comprehensive assessment of the full texts, 14 studies met all inclusion criteria and were incorporated into the final systematic review. After full-text assessment, nine articles were excluded due to the use of alternative diagnostic tools, such as conventional computed tomography. An additional five articles were excluded because they employed superimposed 3D models for TMJ space evaluation. Three-dimensional models based on superimposition were not considered, as they do not currently represent the standard for assessing joint space dimensions and cannot be directly compared with the classical linear measurement method in CBCT. In addition, one study was excluded due to insufficiently reported results, where only graphical methods were presented without provision of specific numerical data. The inter-reviewer reliability, assessed using kappa statistics (K = 0.883), indicated a significant agreement between the reviewers.

### 3.2. Study Characteristics

The 14 selected studies were published between 2010 and 2024 (Table 2). Four articles were conducted in China [17,18,19,20], two in Germany [21,22], two in South Korea [23,24], and another two in Iran [25,26]. The rest of the studies were from different countries: Chile [27], Brazil [28], Romania [29], and Turkey [30]. In all studies, joint space measurements based on CBCT imaging were performed at least twice—prior to and following orthognathic surgery. Across all studies included in this review, a total of 527 patients were evaluated. Among these, 300 patients had skeletal class II, while 200 patients presented skeletal class III. In one study [30], the skeletal classification of 27 enrolled patients was not specified. In eight studies [17,19,22,24,26,27,28,29], bimaxillary surgery was performed; in three [18,20,30], isolated mandibular osteotomy; and in the remaining three [21,23,25], patients underwent either single- or double-jaw procedures.

### 3.3. Risk of Bias

The risk of bias assessment showed that most studies demonstrated adequate methodological reporting, with the majority addressing key elements such as a clear research question, defined eligibility criteria, and reliable outcome measures. Of the 14 studies, 6 were rated as Good quality and 8 as Fair. The most common methodological limitations were the absence of sample size justification (Q5), incomplete reporting of blinding (Q8), and lack of repeated outcome measures (Q11). No study was rated as Poor. It is also important to note that the overall risk of bias in the studies included in the review may be affected by the exclusion of studies for which the full-text articles were not accessible. The detailed quality assessment is presented in Table 3.

## 4. Discussion

Orthognathic surgeries are recognized as an effective method for the management of craniofacial skeletal deformities. Surgical correction of both class II and class III skeletal malocclusions allows not only for achieving harmonious facial features, but also for obtaining stable occlusion. Moreover, it may reduce TMD symptoms in certain patients after surgery [31] and enhance their quality of life [32]. Over the years, numerous surgical techniques have been introduced to correct skeletal malocclusions, many of which have subsequently undergone refinements. In mandibular osteotomy, various approaches continue to have their proponents, whereas in maxillary osteotomy, the Le Fort osteotomy remains the gold standard in orthognathic surgery. Importantly, repositioning of the maxilla and mandible induces complex anatomical and functional changes, including alterations in muscle tension distribution, which may in turn influence the biomechanics of the TMJ [33]. Nevertheless, the question of whether orthognathic surgery causes changes in the relationship of articular surfaces within the TMJs has not yet been fully resolved. Because of the small sample size in most studies, the aim of this review was to collect all available research meeting the inclusion criteria in order to answer this question.

In 6 out of 14 studies included in this review, joint surface measurements were conducted in both sagittal and coronal planes [18,21,23,25,28,29].

In four of these articles, significant changes were observed in the coronal plane. Vogl et al. [21] reported a decrease in all measured parameters: lateral joint space (LJS) from 2.10 to 1.90/2.30 to 2.04, medial joint space (MJS) from 2.10 to 1.90. A significant decrease was also observed by Da Silva et al. [28], where MJS dropped from 3.80 to 2.93. In the remaining two studies, isolated significant results were noted, showing increases instead. In Roman et al. [29], in class III malocclusion, MJS increased from 2.02 to 2.55. In Zhang et al. [18], LJS in the right TMJ increased from 2.20 to 2.62, and MJS in the left TMJ rose from 1.74 to 2.47.

Regarding sagittal plane measurements, the study by Ravelo et al. [27] is particularly noteworthy, as significant differences were observed in all joint spaces. Six months post-surgery, depending on skeletal class and consequently depending on mandibular advancement or setback during BSSO—distinct changes in joint space were recorded. In class II patients, with mandibular advancement, the anterior joint space (AJS) increased from 1.34/1.48 to 1.74/1.84, while the superior joint space (SJS) decreased from 2.61/2.83 to 1.51/1.52, and the posterior joint space (PJS) from 3.03/2.82 to 2.61/2.37. Thus, the condyle shifted upward and backward relative to the articular fossa. In class III patients, with mandibular setback, AJS decreased from 2.53/2.15 to 1.81/1.68 and SJS from 2.31/1.98 to 1.55/1.82, while PJS increased from 1.45/1.17 to 1.63/1.46, indicating a forward and upward movement of the condyle. These results were partly confirmed by Abbasi et al. [25], who reported a significant AJS increase in class II patients from 2.10 to 2.31, and by Chen et al. [19], who found an increase in AJS from 2.39/2.30 to 3.31/3.33 and a decrease in PJS from 2.66/2.74 to 2.18/2.16.

Roman et al. [29] also noted an AJS increase in class II (2.45 to 2.87), but PJS increased as well from 2.75 to 3.49. A decrease in PJS in class II was reported by Yang et al. [17] from 2.87 to 2.16, and Vogl et al. [21] from 2.20/2.30 to 2.00/1.90. Conversely, increases in class III were found by Kuehle et al. [22] a difference of −0.97/−0.52 compared to preoperative values, and Huang et al. [20] from 1.63/1.85 to 4.18/4.93 and 2.33/2.74.

In agreement with Ravelo et al. [27], significant decreases in SJS were also observed in studies by Yang et al. [17] from 3.09 to 2.75, Vogl et al. [21] from 2.50/2.80 to 2.30/2.60, Da Silva et al. [28] from 2.90 to 2.60, Meral et al. [30] from 3.31 to 2.69, and Kuehle et al. [22] from −0.29/−0.25 and −0.85/−0.65 compared to preoperative values.

Interestingly, in four studies, SJS significantly increased: Zhang et al. [18] from 1.82/1.71 to 2.17/2.24, Chen et al. [19] from 2.97/2.84 to 3.90/3.71, Huang et al. [20] from 2.07/2.19 to 4.73/5.34, and Tabrizi et al. [26] from 3.62 to 4.13.

The only study reporting no significant differences in any joint space was by Han et al. [23], where, despite four measurements, statistical analysis was only performed between pre-treatment results and retention-phase values. However, the authors did not report the precise timing of the CBCT examinations, which implies that the interval between preoperative and postoperative assessments may have ranged from 12 to 18 months. Therefore, the absence of significant results may be linked to observations made by other researchers [19,24,25,26], who suggested that, over time, the condyle may partially return to its preoperative position. Kuehle et al. [22] proposed that postoperative remodeling directs the condylar process back to nearly its original location in the articular fossa.

This is consistent with the findings of other studies, which suggest that this return may be explained by physiologic adaptive bone remodeling [34], occurring as a result of the recovery of oral function after orthognathic surgery, particularly through improvements in neuromuscular function and bite force [35]. Yang et al. [17] emphasized that such functional remodeling of the condyle is regarded as a form of physiological morphological change. Kim et al. [24] further highlighted that physiologic adaptation can compensate for minor alterations in condylar position. And this would be followed either by later skeletal relapse or condylar remodeling, depending on the individual patient’s capacity [24].

There is still no consensus on whether such changes also influence the development of TMD [25,30]. Disc displacement, if within the limits of physiological balance and adaptive capacity, does not cause significant changes and does not lead to TMD [36]. On the contrary, although orthognathic surgery is not considered the main treatment for TMD [35], it may reduce myofascial pain through anatomical corrections [37] and improve the harmony of the stomatognathic system. However, if adaptive capacity is exceeded, condylar resorption and remodeling may occur to such an extent that changes in condylar volume disturb TMJ balance [38]. Such altered harmony may contribute to the appearance of TMD symptoms [36]. It is noteworthy that while orthognathic surgery may lead to symptom improvement in patients with pre-existing TMD, it can also result in the onset of symptoms in asymptomatic individuals [31]. Importantly, neither the type of skeletal deformity nor the preoperative symptom status has demonstrated consistent prognostic value [31]. These findings suggest that CBCT-based structural alterations of the temporomandibular joint may not directly correspond to clinical symptom expression.

It is known that postoperative condylar position can depend on several factors, including rotational movements of the distal segment, surrounding muscle tension, fixation methods, and the surgeon’s experience [39]. Additionally, it may be influenced by preexisting TMD [40], technical issues during surgery [41], the magnitude of setback [42,43], biomechanical stress on the condyle [44], as well as vertical and horizontal skeletal patterns [45,46,47,48,49]. Importantly, disc displacement related to TMD has been associated with decreased SJS in the TMJ [50]. Therefore, in some patients with significant SJS reduction after surgery, the risk of developing TMD symptoms may be higher.

As mentioned earlier, condylar position after surgery is influenced by many variables. Some studies suggest that, although the goal of BSSO and IVRO is the same, IVRO may provide better TMJ outcomes through adaptive remodeling and anteroinferior condylar displacement after surgery [51,52,53].

Several limitations of this review should be acknowledged. First, due to a lack of access to full-text articles, some initially accepted studies had to be excluded from analysis. Also, the exclusion of studies employing 3D model-based methods reduced the overall number of eligible publications; however, including them could have introduced bias, as their measurement methodology is not directly comparable to conventional CBCT-based linear assessments. Another limitation is that follow-up assessments were conducted at different time intervals. While this complicates cross-study comparisons, in articles where postoperative CBCT scans were taken multiple times, it allowed for broader analysis and additional conclusions. Finally, the included studies differed in both surgical techniques and operative methods. Nonetheless, the main purpose of this review was to analyze TMJ changes following all types of orthognathic surgical procedures.

## 5. Conclusions

Orthognathic surgery is associated with measurable changes in TMJ spaces; however, the direction and magnitude of these changes are heterogeneous and dependent on skeletal class, type of procedure, and observation period. The magnitude, direction, and clinical significance of these changes appear to be influenced by multiple factors, including the specific surgical approach and the patient’s individual adaptive capacity. Their clinical significance remains uncertain. The overall certainty of evidence is limited by small sample sizes, heterogeneous study designs, and incomplete reporting. Further high-quality studies are warranted to clarify the long-term implications of these findings for TMJ function and patient outcomes. The results of this analysis support the need for standardization of pre- and postoperative follow-up protocols, as no guidelines currently exist and temporomandibular joint assessment is performed inconsistently.

## Figures and Tables

**Table 1:** Inclusion and exclusion criteria.

Inclusion Criteria	Exclusion Criteria
Studies involved patients undergoing orthognathic surgery, specifically Le Fort I osteotomy, BSSO, and/or IVRO and/or HSSO.	Studies that assessed TMJ space only at a single time measurement (i.e., either preoperative or postoperative only) and/or measured non-comparable anatomical reference points.
Studies that reported TMJ space measurements acquired at least twice, both preoperatively and postoperatively.	Studies that used methods relying on superimposed 3D models for TMJ space evaluation.
Studies that assessed joint space dimensions in the sagittal and/or coronal planes, using consistent anatomical reference points to obtain measurements of anterior, posterior, superior, medial, or lateral joint spaces.	Pilot studies, ex vivo studies, cross-sectional studies, review articles, pre-prints, editorials, conference abstracts.
Retrospective pre–post studies.	The full text of the article was not accessible.
The full-text is accessible.	

**Table 2:** Data extracted from included studies.

Author, Study Location, and Year of Publication	Sample Size–Skeletal Class, Age, Gender	Type of Procedure	CBCT Parameters	Time of CBCT Before/After	Preoperative Measurements in the Sagittal Plane (mm)		Postoperative Measurements in the Sagittal Plane (mm)			*p*-Value	Preoperative Measurements in the Coronal Plane (mm)		Postoperative Measurements in the Coronal Plane (mm)			*p*-Value
Kim et al., South Korea, 2010 [24]	26 patients 26 class III 14 females 12 males mean age: 21.30 ± 4.38	26 BSSO + Le Fort I	DCT Pro (Vatech, Seoul, Republic of Korea) 20 × 19 cm field of view, 90 kVp, 4 mA	T0 presurgery T1 postsurgery 6 months T2 follow-up 18 months	T0 AJS (R) 1.81 AJS (L) 1.68 SJS (R) 2.67 SJS (L) 2.67 PJS (R) 2.43 PJS (L) 2.69		T1 AJS (R) 2.40 AJS (L) 2.25 SJS (R) 2.70 SJS (L) 2.68 PJS (R) 2.28 PJS (L) 2.18	T2 AJS (R) 1.88 AJS (L) 1.92 SJS (R) 2.42 SJS (L) 2.69 PJS (R) 2.25 PJS (L) 2.44		0.04 * 0.01 * 0.46 0.99 0.70 0.23	NR		NR			NR
Chen et al., People’s Republic of China, 2013 [19]	27 patients 27 class II mean age: 27.0 ± 5.4	27 BSSO + Le Fort I	DCT Pro (Vatech, Seoul, Republic of Korea) 16 × 10 cm field of view, 90 kVp, 7.0 mA	T0 1 week before T1 3–5 days after surgery T2 3 months after T3 12 ± 3 month after	T0 AJS (R) 2.39 AJS (L) 2.20 SJS (R) 2.97 SJS (L) 2.84 PJS (R) 2.79 PJS (L) 2.71		T1 AJS (R) 3.31 AJS (L) 3.33 SJS (R) 3.90 SJS (L) 3.71 PJS (R) 2.66 PJS (L) 2.74	T2 AJS (R) 2.35 AJS (L) 2.24 SJS (R) 2.51 SJS (L) 2.41 PJS (R) 2.18 PJS (L) 2.16	T3 AJS (R) 2.39 AJS (L) 2.23 SJS (R) 2.46 SJS (L) 2.34 PJS (R) 2.17 PJS (L) 2.10t	T1–T0 *p* < 0.05 T1–T0 *p* < 0.05 T1–T0 *p* < 0.05 T2–T1 *p* < 0.05 T2–T0 *p* < 0.05 T1–T0 *p* < 0.05 T2–T1 *p* < 0.05 T2–T0 *p* < 0.05 T2–T1 *p* < 0.05 T2–T1 *p* < 0.05	NR		NR			NR
Kuehle et al., Germany, 2016 [22]	50 patients 24 class II 26 class III 32 females 18 males mean age: 26.3 ± 7.4	50 HSSO + Le Fort I	Gallileos Comfort plus system (Sirona Dental Systems GmbH, Bensheim, Germany) 15.4 cm × 0.125 mm for each voxel, 98 kV, 3–6 mA	two weeks preoperatively/2–4 days postop- eratively/9 months	changes after surgery CLASS II AJS (R) -0.52 AJS (L) −0.49 SJS (R) −0.29 SJS (L) −0.25 PJS (R) −0.43 PJS (L) −0.33 CLASS III AJS (R) −0.29 AJS (L) −0.52 SJS (R) −0.85 SJS (L) −0.65 PJS (R) −0.97 PJS (L) −0.52		changes after 9-month follow-up CLASS II AJS (R) 0.03 AJS (L) 0.03 SJS (R) 0.78 SJS (L) 0.68 PJS (R) 0.88 PJS (L) 0.78 CLASS III AJS (R) 0.10 AJS (L) −0.07 SJS (R) −0.01 SJS (L) 0.36 PJS (R) 0.02 PJS (L) 0.19			0.20 0.21 0.02 * 0.04 * 0.00 * 0.05 * 0.3 0.4 0.00 * 0.00 * 0.00 * 0.01 *	NR		NR			NR
Tabrizi et al., Iran, 2016 [26]	22 patients 22 class II 25 females 7 males mean age: 22.18 ± 5.6	22 BSSO + Le Fort I	New Tom VGI Flex (Image work Co.; White Plains, NY, USA) 15 × 15 cm with 0.3 mm slice	T0 couple days before surgery T1 one month after surgery T2 9 months after surgery	SJS 3.62 (T0) SJS 3.62 (T0) SJS 4.13 (T1)		SJS 4.13 (T1) SJS 3.80 (T2) SJS 3.80 (T2)			*p* < 0.05 * p > 0.05 *p* > 0.05	NR		NR			NR
Da Silva et al., Brazil, 2018 [28]	57 patients 57 class II 40 females 17 males mean age: 31.4	57 BSSO + Le Fort I	i-CAT Next Generation scanner (Imaging Sciences International, Hatfield, PA, USA) 120 kVp, 5 mA, 23 × 17 cm field of view, 0.4 mm voxel size	Pre-surgical/at least 6 months	AJS 3.5 SJS 2.9 PJS 3.1		AJS 3.6 SJS 2.6 PJS 2.92			0.39 0.00 * 0.12	MJS 3.8		MJS 2.93			<0.00 *
Zhang et al., China, 2018 [18]	10 patients 10 class III 5 females 5 males mean age: 25.0	10 BSSO	CT (KaVo 3D eXam) 120 kVp, 3–8 mA, 0.4 mm	6 months before/6 months after	AJS (R) 2.71 AJS (L) 2.54 SJS (R) 1.82 SJS (L) 1.71 PJS (R) 2.34 PJS (L) 2.66		AJS (R) 2.70 AJS (L) 2.53 SJS (R) 2.17 SJS (L) 2.24 PJS (R) 2.56 PJS (L) 2.52			*p* > 0.05 *p* > 0.05 *p* < 0.01 * p < 0.01 * p > 0.05 *p* > 0.05	LJS (R) 2.20 LJS (L) 2.86 MJS (R) 2.27 MJS (L) 1.74		LJS (R) 2.62 LJS (L) 2.69 MJS (R) 2.41 MJS (L) 2.47			*p* < 0.05 * p > 0.05 *p* > 0.05 *p* < 0.01 *
Huang et al., China, 2020 [20]	21 patients 21 class III 11 females 10 males Age: 18–33	21 IVRO	NewTom scanner (Imaging Science International, Hatfield, PA, USA) field of view: 200 × 400 mm, 120 kVp, 47.7 mA, 0.4 mm voxel size	T0 before surgery T1 one week after surgery T2 6 months after surgery	T0 AJS (R) 1.56 AJS (L) 1.45 SJS (R) 2.07 SJS (L) 2.19 PJS (R) 1.63 PJS (L) 1.85		T1 AJS (R) 1.18 AJS (L) 1.14 SJS (R) 4.73 SJS (L) 5.34 PJS (R) 4.18 PJS (L) 4.93	T2 AJS (R) 1.61 AJS (L) 1.50 SJS (R) 2.72 SJS (L) 3.26 PJS (R) 2.33 PJS (L) 2.74		NS NS 0.00 * 0.00 * 0.00 * 0.00 *	NR		NR			NR
Roman et al., Romania, 2022 [29]	28 patients 14 class II 14 class III 19 females 9 males mean age: 26.85 ± 6.54 (class II) mean age: 26.64 ± 6.87 (class III)	28 BSSO + Le Fort I	Promax 3D Max (Planmeca, Helsinki, Finland) 23 × 23 × 16 cm, 0.4 mm voxel size, 86–88 kV, 6–8 mA	T0 before surgery T1 two days after surgery	CLASS II AJS 2.45 PJS 2.75 CLASS III AJS 2.15 PJS 2.01		CLASS II AJS 2.87 PJS 3.49 CLASS III AJS 2.00 PJS 2.16			0.02 * 0.01 * 0.14 0.5	CLASS II MJS 2.98 CLASS III MJS 2.02		CLASS II MJS 2.91 CLASS III MJS 2.55			0.41 0.01 *
Han et al., Republic of Korea, 2023 [23]	26 patients 26 class III 15 females 11 males mean age: 19.6 ± 2.8	10 BSSO 16 BSSO + Le Fort I	PSR 9000N (Asahi Alphard Vega, Kyoto, Japan) C-mode: Scan size 2003 179 mm; voxel size 0.39 mm; field of view 19.97 cm	T0 pretreatment T1 presurgery T2 postsurgery T3 posttreatment and/or T4 retention (at least 1 year posttreatment)	T0 AJS 1.43 SJS 2.21 PJS 1.56	T1 AJS 1.57 SJS 2.27 PJS 1.53	T2 AJS 1.78 SJS 2.30 PJS 1.52	T3 AJS 1.60 SJS 2.31 PJS 1.53	T4 AJS 1.64 SJS 2.22 PJS 1.57	0.27 0.98 0.99	T0 MJS 1.96 CJS 2.01 LJS 1.50	T1 MJS 1.88 CJS 2.04 LJS 1.49	T2 MJS 2.30 CJS 2.07 LJS 1.54	T3 MJS 2.08 CJS 2.01 LJS 1.55	T4 MJS 2.09 CJS 2.25 LJS 1.75	0.20 0.82 0.58
Meral et al., Turkey, 2023 [30]	27 patients (NR)	27 BSSO	i-CAT Next Generation scanner (Imaging Sciences International, Hatfield, PA, USA) 120 kVp, 5–7 mAs, 23 × 17 cm field of view, 0.3 mm voxel size	NR	CONTROL GROUP SJS 2.24 AJS 2.26 PJS 1.87 CONTRALATERAL GROUP SJS 2.67 AJS 2.29 PJS 2.73 DEVIATION GROUP SJS 3.31 AJS 2.75 PJS 2.50		CONTROL GROUP SJS 2.30 AJS 2.58 PJS 2.12 CONTRALATERAL GROUP SJS 2.49 AJS 2.19 PJS 2.55 DEVIATION GROUP SJS 2.69 AJS 2.74 PJS 2.21			0.64 0.05 * 0.10 0.57 0.59 0.83 0.05 * 0.86 0.36	NR		NR			NR
Ravelo et al., Chile, 2023 [27]	26 patients 15 class II 11 class III 14 females 12 males mean age: 27.9 ± 10.81	BSSO + Le Fort I	NewTom 3D software, Vgi EVO model (Verona, Italy) 24 × 19 cm field of view, 110 kV, 8 mA	21 days prior to the surgery/6 months after	CLASS II AJS (R) 1.34 AJS (L) 1.48 SJS (R) 2.61 SJS (L) 2.83 PJS (R) 3.03 PJS (L) 2.82 CLASS III AJS (R) 2.53 AJS (L) 2.15 SJS (R) 2.31 SJS (L) 1.98 PJS (R) 1.45 PJS (L) 1.17		CLASS II AJS (R) 1.74 AJS (L) 1.84 SJS (R) 1.51 SJS (L) 1.52 PJS (R) 2.61 PJS (L) 2.37 CLASS III AJS (R) 1.81 AJS (L) 1.68 SJS (R) 1.55 SJS (L) 1.82 PJS (R) 1.63 PJS (L) 1.46			CLASS II 0.00 * 0.00 * 0.00 * CLASS III 0.00 * 0.00 * 0.02 *	NR		NR			NR
Yang et al., Republic of China, 2023 [17]	97 patients 97 class II 77 females 20 males mean age: 24.82	97 BSSO + Le Fort I + genioplasty	LCBCT unit (Morita Corp., Hyogo, Japan) 80 kV at 4.5 mA pulse operation	before orthodontics/12 months after surgery	AJS 1.68 SJS 3.09 PJS 2.87		AJS 1.68 SJS 2.75 PJS 2.16			0.96 0.05 * 0.00 *	NR		NR			NR
Abbasi et al., Iran, 2024 [25]	31 patients 15 class II 16 class III	8 BSSO 23 BSSO + Le Fort I	Acteon Whitefox CBCT scanner 80 KVP, 5 mA, 0.3 mm voxel size, field of view 170 × 200	before surgery/min 6 months after surgery	CLASS II SJS 2.46 AJS 2.10 PJS 3.04 CLASS III SJS 2.52 AJS 2.01 PJS 3.00		CLASS II SJS 2.46 AJS 2.31 PJS 2.94 CLASS III SJS 2.52 AJS 2.13 PJS 2.89			0.18 0.01 * 0.01 * 0.48 0.20 0.01 *	CLASS II MJS 2.87 LJS 3.63 CLASS III MJS 2.81 LJS 3.40		CLASS II MJS 2.84 LJS 3.62 CLASS III MJS 2.87 LJS 3.41			0.41 0.46 0.48 0.60
Vogl et al., Germany, 2024 [21]	79 patients 29 class II 50 class III 41 females 38 males mean age: 26.62 ± 9.5	19 BSSO 60 BSSO + Le Fort I	PLANMECA ProMax-3D Max CBCT device (Planmeca Oy, Helsinki, Finland) field of view: 230 mm/160 mm 120 kV and 7.1 mA	Before surgery/after surgery with a median of 6 weeks	AJS (R) 2.10 AJS (L) 2.10 SJS (R) 2.50 SJS (L) 2.80 PJS (R) 2.20 PJS (L) 2.30		AJS (R) 2.20 AJS (L) 2.10 SJS (R) 2.30 SJS (L) 2.60 PJS (R) 2.00 PJS (L) 1.90			0.13 0.26 0.01 * 0.01 * 0.01 * 0.00 *	LJS (R) 2.10 LJS (L) 2.30 MJS (R) 2.10 MJS (L) 2.10		LJS (R) 1.90 LJS (L) 2.04 MJS (R) 1.90 MJS (L) 1.90			0.02 * 0.01 * 0.00 * 0.01 *

* *p* is significant at the 0.05 level; AJS—anterior joint space; SJS—superior joint space; —posterior joint space; MJS—medial joint space; LJS—lateral joint space; L—left; R—right; NR—not reported.

**Table 3:** Risk of bias.

Study (Author, Year)	Q1	Q2	Q3	Q4	Q5	Q6	Q7	Q8	Q9	Q10	Q11	Q12	Overall Rating
Kim et al., 2010 [24]	Yes	Yes	No	Yes	No	Yes	Yes	NR	Yes	Yes	Yes	NA	Fair
Chen et al., 2013 [19]	Yes	Yes	Yes	Yes	No	Yes	Yes	Yes	Yes	Yes	Yes	NA	Good
Kuehle et al., 2016 [22]	Yes	Yes	Yes	Yes	No	Yes	Yes	NR	Yes	Yes	Yes	NA	Good
Tabrizi et al., 2016 [26]	Yes	Yes	Yes	Yes	No	Yes	Yes	NR	Yes	Yes	Yes	NA	Good
Da Silva et al., 2018 [28]	Yes	Yes	Yes	Yes	No	Yes	Yes	NR	Yes	Yes	No	NA	Fair
Zhang et al., 2018 [18]	Yes	Yes	Yes	Yes	No	Yes	Yes	NR	Yes	Yes	No	NA	Fair
Huang et al., 2020 [20]	Yes	No	Yes	Yes	No	Yes	Yes	NR	Yes	Yes	Yes	NA	Fair
Roman et al., 2022 [29]	Yes	Yes	Yes	Yes	No	No	Yes	NR	Yes	Yes	No	NA	Fair
Han et al., 2023 [23]	Yes	Yes	Yes	Yes	No	No	Yes	Yes	Yes	Yes	Yes	NA	Fair
Meral et al., 2023 [30]	Yes	Yes	Yes	Yes	No	No	Yes	Yes	Yes	Yes	Yes	NA	Fair
Ravelo et al., 2023 [27]	Yes	Yes	Yes	Yes	No	Yes	Yes	Yes	Yes	Yes	No	NA	Good
Yang et al., 2023 [17]	Yes	Yes	Yes	Yes	No	Yes	Yes	Yes	Yes	Yes	No	NA	Good
Abbasi et al., 2024 [25]	Yes	Yes	Yes	Yes	Yes	Yes	Yes	NR	Yes	Yes	No	NA	Good
Vogl et al., 2024 [21]	Yes	Yes	Yes	Yes	No	No	Yes	Yes	Yes	Yes	No	NA	Fair

NR: not reported, NA: not applicable; Good: ≥9 “Yes,” no critical flaws, Fair: 5–8 “Yes,” ≤1 critical flaw, Poor: <5 “Yes” OR ≥2 critical flaws.

## Data Availability

The raw data supporting the conclusions of this article will be made available by the authors on request.

## Supplementary Materials

The following supporting information can be downloaded at: https://www.mdpi.com/article/10.3390/jcm14207239/s1, Table S1: Search strategies.

## Abbreviations

The following abbreviations are used in this manuscript:

TMJ	Temporomandibular joint
TMDs	Temporomandibular disorders
AJS	Anterior joint space
SJS	Superior joint space
PJS	Posterior joint space
LJS	Lateral joint space
MJS	Medial joint space
OSA	Obstructive sleep apnea
BSSO	Bilateral sagittal split osteotomy
IVRO	Intraoral vertical ramus osteotomy
HSSO	High oblique sagittal split osteotomy
