# Supplementary material for: Alterations in the Temporomandibular Joint Space Following Orthognathic Surgery Based on Cone Beam Computed Tomography: A Systematic Review
Source: J Clin Med. 2025 Oct 14;14(20):7239. doi: 10.3390/jcm14207239 (PMC12564798; doi:10.3390/jcm14207239)
Supplement: Supplementary file 1 [file jcm-14-07239-s001.zip › jcm-3893973-supplementary.pdf]

Table S1: Search strategies

Database	Query	Field	Records retrieved
PubMed	((((((("temporomandibular joint") OR (TMJ)) AND (Space)) AND ("orthognathic surgery")) OR ("Le fort I")) OR ("bilateral sagittal split osteotomy")) OR ("Intraoral vertical ramus osteotomy")) OR ("surgical orthodontic treatment")) AND (change)	All	1,014
Cochrane (Wiley)	(temporomandibular joint OR TMJ):ti,ab,kw AND (Space):ti,ab,kw AND (orthognathic surgery OR bilateral sagittal split osteotomy OR Intraoral vertical ramus osteotomy OR surgical orthodontic treatment):ti,ab,kw AND (change):ti,ab,kw	Title/Abstract /Keywords	3
Web of Science	((((((("temporomandibular joint") OR (TMJ)) AND (Space)) AND ("orthognathic surgery")) OR ("Le fort I")) OR ("bilateral sagittal split osteotomy")) OR ("Intraoral vertical ramus osteotomy")) OR ("surgical orthodontic treatment")) AND (change)	All	1,047
Scopus	(ALL ("temporomandibular joint") OR ALL (tmj) AND ALL (space) AND ALL ("orthognathic surgery") OR TITLE-ABS-KEY ("Le fort I") OR ALL ("bilateral sagittal split osteotomy") OR ALL ("Intraoral vertical ramus osteotomy") OR ALL ("surgical orthodontic treatment") AND ALL (change))	All	697

Searched databases: PubMed, Web of Science, Cochrane (Wiley), and Scopus
Searching Date: 09-04-2025
No filters applied, and no publication date restrictions.
